# Social Connectedness and Successful Nursing Home Discharge After Heart Failure Hospitalization

**DOI:** 10.1016/j.jamda.2025.105824

**Published:** 2025-09-02

**Authors:** Andrew L. Chen, Blythe G. Chen, Lan Jiang, Matthew Howe, Matthew F. Thompson, Julia Browne, Zachary J. Kunicki, John McGeary, James L. Rudolph, Thomas A. Bayer

**Affiliations:** a The Warren Alpert Medical School of Brown University, Providence, RI, USA; b Brown University School of Public Health, Providence, RI, USA; c Center of Innovation on Transformative Health Systems Research to Improve Veteran Equity and Independence (THRIVE COIN), VA Providence Healthcare System, Providence, RI, USA; d Brown University Department of Psychiatry and Human Behavior, Providence, RI, USA; e Division of Geriatrics and Palliative Medicine, The Warren Alpert Medical School of Brown University, Providence, RI, USA

**Keywords:** Social connectedness, successful discharge, heart failure

## Abstract

**Objectives:**

Social connectedness is associated with positive health outcomes. Patients discharged to skilled nursing facilities (SNFs) after heart failure (HF) hospitalization face a high risk of hospital readmission, but the association between social connectedness and successful discharge from postacute SNF care is unknown. This study aimed to quantify the association between social connectedness and successful discharge from postacute SNF care among veterans with HF.

**Design:**

This retrospective cohort study’s primary outcome was successful discharge, defined as discharge to the community within 90 days of admission to the SNF, and survival 30 days after discharge without hospitalization or institutionalization. Social connectedness was measured by the Social Connectedness Index [SCI, range 0–5: binarized into low (SCI = 0–4) or high social connectedness (SCI = 5)].

**Setting and Participants:**

Veterans admitted to a Department of Veterans Affairs Medical Center for HF and subsequently discharged to a SNFs between January 2011 and June 2019.

**Methods:**

We estimated the association of high SCI with successful discharge using a modified Poisson regression with robust error variance.

**Results:**

A total of 29,725 veterans were included. Veterans with high social connectedness (SCI = 5) in SNF settings were more likely to have successful discharge than those with lower social connectedness [adjusted relative risk (95% CI): 1.21 (1.13–1.31)]. This association was seen in patients with Alzheimer disease or Alzheimer disease and related dementias (AD/ADRD) [1.32 (1.16–1.49)] and without ADRD [1.14 (1.04–1.25)] cohorts.

**Conclusions and Implications:**

Veterans with HF who were more socially connected in the SNF setting had higher rates of successful discharge than those with lower social connectedness. Low social connectedness may be an indicator of care needs that make discharge from SNF to home more challenging. Clinical social isolation measurement may be a useful tool in identifying successful discharge candidates.

Patient-centered care requires balancing the potential for institutionalization to efficiently meet care needs with the desire to reside at home. Qualitative research has found that veterans using geriatrics clinics and home-based services view days at home more favorably than days spent in health care settings when assessing quality of life.^1^ Patients who spend less time in the community (“time at home”) have poorer patient-centered outcome measures, such as limited social activity, difficulty in activities of daily living, impaired mobility, and increased frailty.^2,3^ Although optimization of time at home is an important goal, there are cases in which discharge home is inadvisable, and the needs of patients are better met with nursing home care.

Nursing home care needs are measured in terms of activities of daily living and mobility but needs related to social behavior are less well understood as indicators of nursing home need. Older adults are particularly vulnerable to the risks of loneliness and social isolation, which are associated with a wide range of negative health impacts, including increased dementia risk and all-cause mortality.^4–6^ Loneliness and social isolation are commonly measured using self-report questionnaires,^7^ and are sometimes measured using one question such as “Do you ever feel lonely?” These scales may be limited by reporting or social desirability bias, and have wide variations in prevalence estimates across studies in the nursing home setting.^8^ The Social Connectedness Index (SCI) characterizes social well-being in the nursing home setting using routinely collected patient data to estimate social connectedness vs social disengagement.^9^ Social connectedness is defined as the relationships that people have with their social networks, greater communities, and immediate environment, and social disengagement arises from a lack of social connectedness.^9^ The SCI was originally developed for patients with Alzheimer disease and Alzheimer disease and related dementias (AD/ADRD) and is calculated by assessing a mix of observable behaviors captured in the MDS dataset—not self-perceived isolation. The SCI defines social connectedness as the absence of behaviors that interfere with care and social interactions within the nursing home setting.^9^ This definition of social connectedness differs from self-report measures of social isolation that include items regarding the user’s outside support networks. The SCI focuses solely on observable behaviors within the nursing home, making it uniquely suited for measuring a patient’s ability to form social connections within the nursing home setting and identifying behaviors that are barriers to social connection. This provides an opportunity for caregivers to detect patients who are at risk of social disengagement within nursing homes and potentially mitigate the negative health consequences associated with social disengagement.

We hypothesized that higher levels of social connectedness would be associated with successful discharge home from skilled nursing facilities (SNFs) after heart failure (HF) admissions. We studied HF hospitalization as a model condition, as it is known for high rates of unplanned postacute readmission and heavy reliance on self-management.^10–15^ Social connectedness is particularly important in the context of HF, as poor connectedness and has been associated with increased risk of death, hospitalization, and emergency department visits in patients with HF living in the community.^16^ Thus, a patient’s level of social connectedness may play a key role in identifying those at risk for unsuccessful discharge home. We designed a study to estimate the association between social connectedness and successful discharge home from SNF after HF admissions in a cohort of veterans.

## Methods

### Data

We completed an observational retrospective cohort study to estimate the effects of social connectedness on successful discharge from SNF in veterans after HF hospitalization between January 2011 and June 2019. We obtained baseline clinical and demographic data from the VA Corporate Data Warehouse (CDW) and successful discharge data from linked VA and Centers for Medicare & Medicaid Services (CMS) data sources, such as the Minimum Data Set 3.0 (MDS).

### Participants

Veterans who were hospitalized with HF and were admitted to an SNF within 7 days of hospital discharge were included in the analysis. For veterans with multiple HF-related admissions, one hospitalization was randomly selected to reduce bias toward either the first or last hospitalization. Before selection of a random admission, additional exclusions were applied: veterans were excluded if they were in the hospital, community living center, nursing home, or SNF within 30 days before indexed hospital admission, indicating unsuccessful discharge, or if they did not have an MDS admission assessment containing the items required to calculate the SCI indicating incomplete or missing data. Approval for this study was obtained from the local institutional review board that waived informed consent for this secondary analysis.

### Outcome

The primary outcome in this study was successful discharge, defined as discharge to the community within 90 days of SNF admission where the veteran was alive 30 days after discharge without rehospitalization or institutionalization. Discharges were not considered successful if the discharge type was reported as hospice in the MDS, as hospice has a different goal from rehabilitation and this fell under our definition of institutionalization. We chose the 30-day follow-up period as it mirrors the CMS measure of successful discharge from SNF to home or community.^17^

### Social Connectedness

Using MDS 3.0 data completed by nursing staff at intake and then at intervals based on length of stay, we calculated the SCI, a 5-point index developed to characterize social connection among nursing home residents with AD/ADRD.^4,9,18^ The SCI includes behavioral symptoms, behaviors interfering with social interactions, behaviors impacting others, wandering intruding on others, and rejection of care, all of which are scored as being either present or absent by nursing home staff. We used the SCI for a mixed-case veteran population, with both AD/ADRD and non-ADRD participants, because SCI can be calculated on behaviors captured in all resident assessments and is focused on characterizing ability to connect on an interpersonal level.^9^ We treated the SCI as a binary variable, classifying veterans as having either lower (SCI = 0–4) or high social connectedness (SCI = 5). These cutoffs were chosen based on previous work with the SCI that used the same cutoffs.^18^ Evidence from previous research suggests that people with high baseline loneliness scores are more likely to be admitted to SNFs, and that social connection dysfunction prevalence in SNFs is quite high.^4^ Therefore, we set a high threshold for social connectedness based on SNF population characteristics and previous study methods.^4,18^

### Covariates

Covariates included basic demographic (age, race, sex) and clinical (medical history, prior year HF admissions, AD/ADRD status, cognitive function, patient-reported pain, 6-month life expectancy) variables. We measured AD/ADRD status using diagnostic codes from the Veterans Health Administration Dementia Diagnostic Codes List (Supplementary Tables 1 and 2).^19^ Individual Elixhauser comorbidities were obtained using International Classification of Diseases (ICD-9 and ICD-10) codes from the VA CDW.^20^ We used the MDS Cognitive Function Scale (CFS) to categorize patients as cognitively intact, or mildly, moderately, or severely impaired using items from the MDS.^21^ We defined ejection fraction (EF) categories of <40%, 40%–−50%, and ≥50%, and binarized smoking to current or former smoker and never smoker. We included pain levels in our analysis because moderate to severe pain can have significant impacts on physical function and quality of life, which can impact one’s ability or willingness to engage socially. We binarized patient-reported pain levels within the MDS 3.0 as no or mild pain and moderate to severe pain.^22^ Six-month life expectancy was a binary variable from the MDS defined as life expectancy of less than 6 months or more than 6 months. This variable requires a physician’s documentation that based on the resident’s diagnosis or clinical conditions, they would not be expected to survive past 6 months.^23^

### Statistical Analysis

We estimated the association of high SCI with successful discharge using a modified Poisson regression with robust error variance.^24^ We used modified Poisson regression because it estimates relative risk, a measure of association familiar to clinicians, and because of its favorable convergence properties compared with other methods of estimating relative risk.^25,26^ We adjusted for confounding variables including age, race, sex, individual Elixhauser comorbidities, EF category, smoking status, prior year HF admissions, prior year total VA encounter cost, MDS CFS score, patient-reported pain levels, and 6-month life expectancy. We then calculated the mean, standard deviation, and relative risk (RR) with 95% confidence intervals (CIs) for our primary outcome. Secondary statistical analysis was an adjusted and unadjusted RR of successful discharge, stratified by AD/ADRD diagnosis. Statistical analysis was performed using Microsoft SQL Server Management Studio version 18 (Microsoft Corporation) and SAS Enterprise Guide version 8.3 (SAS Institute).

## Results

### Demographics

**Fig. 1.**
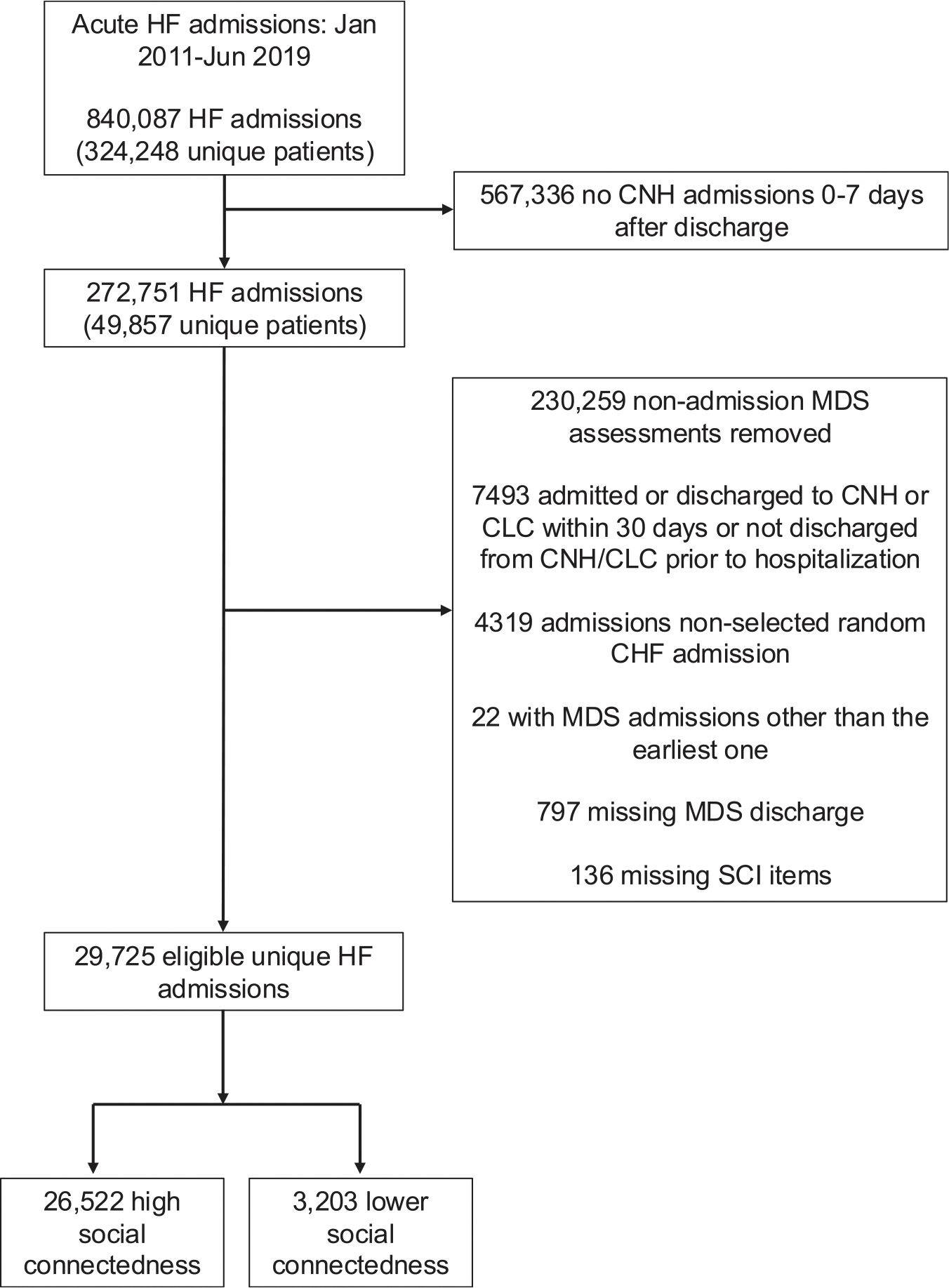
Flow diagram of the study sample. CNH, Community Nursing Home; CLC, Community Living Center.

After exclusion criteria were applied, a total of 29,725 veterans were included in the study (Figure 1). The mean age (SD) at the time of SNF admission was 77.7 (10.3) years old. 28,946 (97.4%) of the cohort identified as male, and 21,984 (73.8%) were White, 5604 (18.9%) were Black, and 2109 (7.1%) were Hispanic (Table 1). A total of 9962 (33.5%) of the cohort had ADRD.

### Social Connectedness

**Fig. 2.**
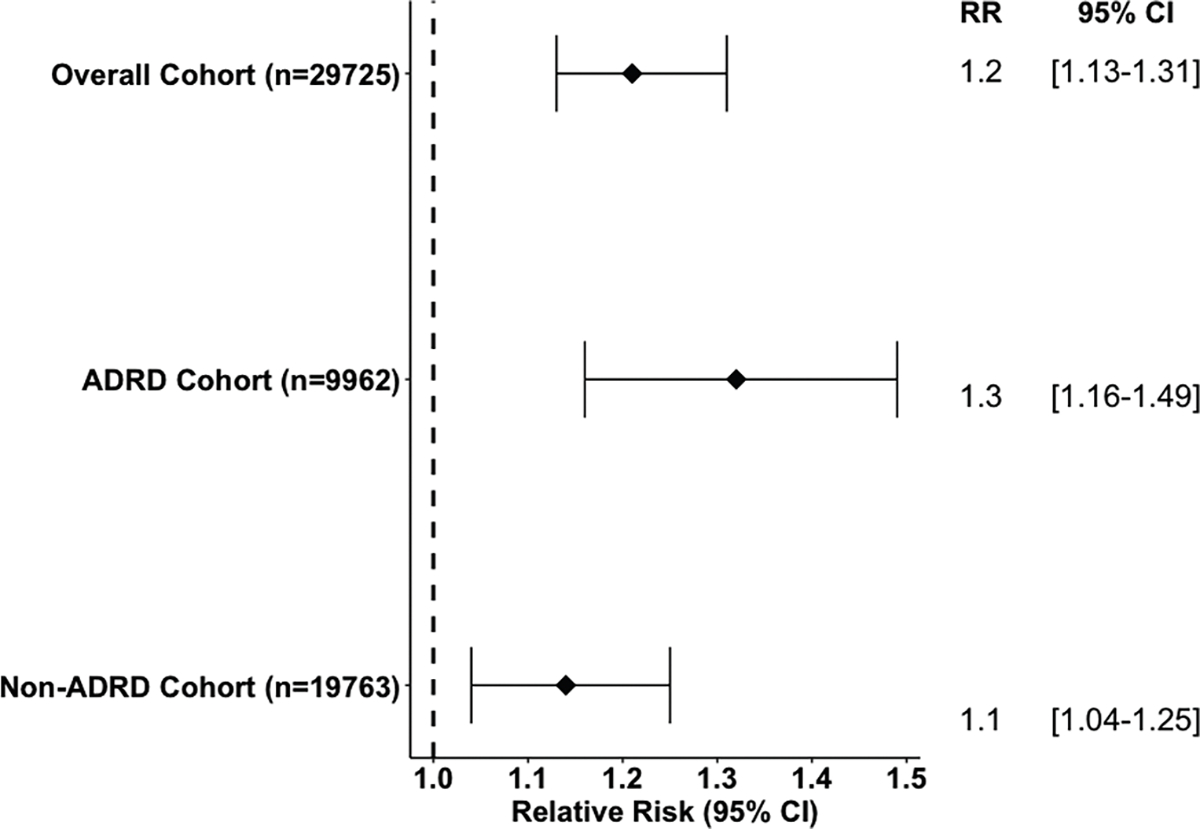
RR (95% CI) of successful discharge associated with successful social connectedness.

Of the 29,725 veterans, 26,522 (89.2%) had high social connectedness (SCI = 5), whereas 3203 (10.8%) had lower social connectedness (SCI = 0–4). The high social connectedness group had a greater proportion of veterans with intact cognitive function per the MDS 3.0 Cognitive Function Scale (62.0%) compared with the low social connectedness group (39.2%). In the overall cohort, the adjusted RR (95% CI) of successful discharge was 1.21 (1.13–1.31) for high vs lower SCI. In veterans with AD/ADRD, the RR of successful discharge was 1.32 (1.16–1.49) with higher social connectedness compared with lower (Figure 2). In veterans without diagnosed AD/ADRD, the RR of successful discharge was 1.14 (1.04–1.25) with higher social connectedness compared with lower.

### Discharge Outcomes

**Fig. 3.**
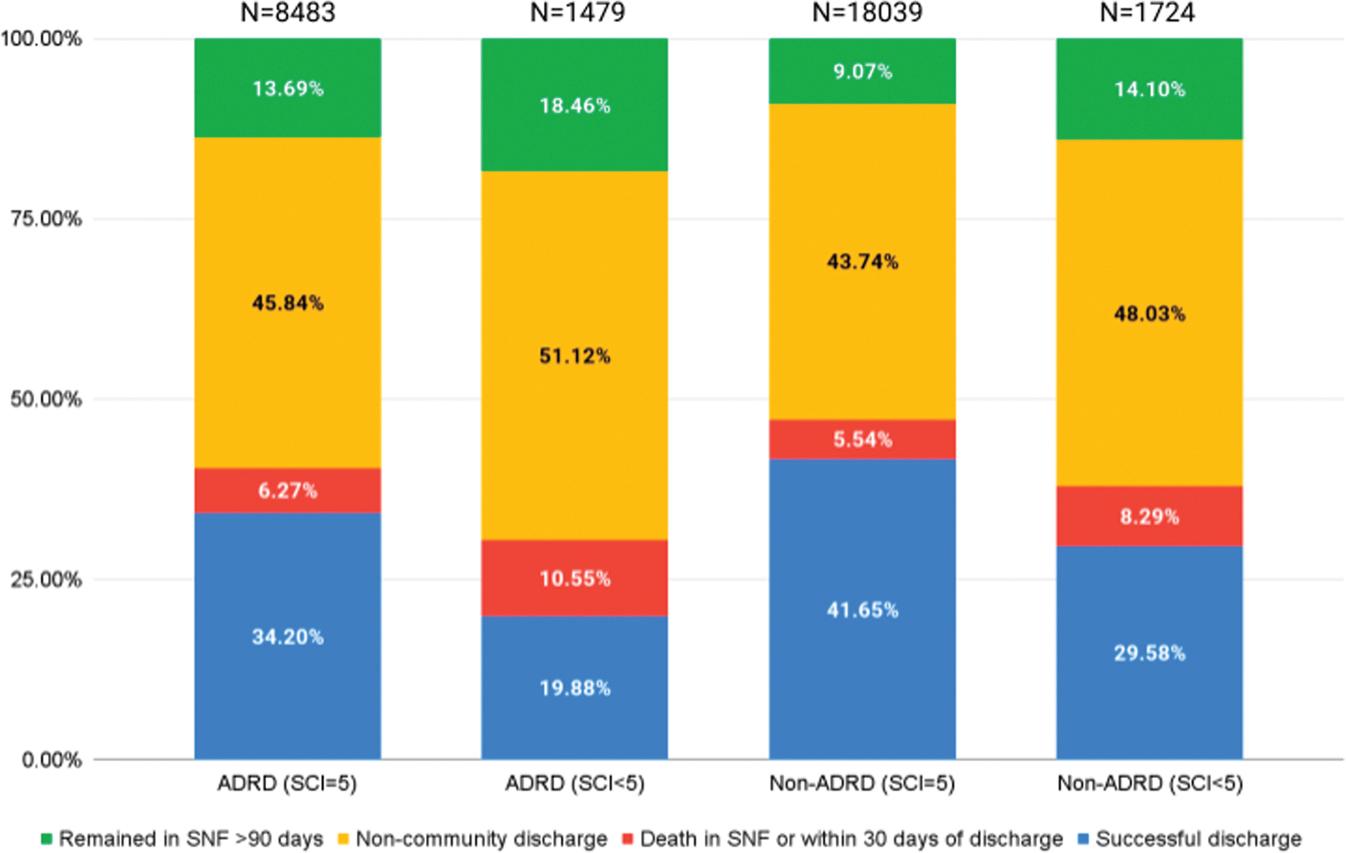
Discharge outcomes.

There were 11,218 successful discharges (37.7%). Of these, 10,414 (92.8%) had high social connectedness, whereas 804 (7.2%) had lower social connectedness. Unsuccessful discharge outcomes included death in the SNF within 90 days (n = 1627), death after discharge home within 30 days (n = 203), no discharge from the SNF after 90 days (n = 3314), noncommunity discharge (n = 6956), and readmission within 30 days after discharge to the community (n = 6407). We compared the percentage of unsuccessful discharge outcomes between high and lower social connectedness (Figure 3). Compared with high social connectedness, lower social connectedness in the AD/ADRD and non-AD/ADRD cohorts was associated with higher rates of remaining in the SNF after 90 days (18.5% vs 13.7% for ADRD, 14.1% vs 9.1% for non-ADRD), noncommunity discharge (51.1% vs 45.8%, 48.0% vs 43.7%, respectively), and death in the SNF or within 30 days of discharge (6.3% vs 10.6%, 8.3% vs 5.5%, respectively).

## Discussion

In this retrospective cohort study, we found that higher levels of social connectedness were associated with successful discharge from postacute SNF care in veterans hospitalized for HF. Successful discharge was defined as discharge to the community within 90 days of SNF admission, where the veteran was alive 30 days after discharge without rehospitalization or institutionalization. This association remained significant after adjusting for baseline characteristics and was seen in AD/ADRD and non-AD/ADRD cohorts. When holding baseline characteristics including age, comorbidity, EF, and cognitive function constant, higher social connectedness remained associated with successful community discharge. This finding was consistent across AD/ADRD and non-ADRD populations, indicating that the SCI, although developed to measure social connectedness in nursing home residents with AD/ADRD retrospectively, has similar prognostic significance in residents without AD/ADRD. To our knowledge, this is the first study to apply SCI to both AD/ADRD and non-ADRD populations.

Our study adds details regarding HF and AD/ADRD to the well-established link between social isolation and poor health outcomes in persons with heart disease and in persons residing in long-term care settings.^4,27^ In a systematic review on social support and health service utilization and survival after cardiovascular disease events in patients from Australia and New Zealand, better social health was associated with discharge to higher independent living, lower rehospitalization, and increased survival.^27^ Our findings align with a body of previous research linking adverse HF outcomes, nonadherence to HF self-management, and social isolation.^28–30^ A recent analysis of UK BioBank data found that social isolation and loneliness were associated with incident HF in a dose-dependent manner independent of genetic HF risk, demonstrating that HF, as well as its complications, may be a consequence of social isolation.^31^ Social isolation was listed as one of the potentially modifiable risk factors from the updated Lancet Commission on dementia, and as of October 2023, the MDS 3.0 now contains an item (D0700) evaluating a resident’s perceived level of social isolation.^32,33^ This newly implemented item highlights the growing interest and utility of using tools such as the SCI in prognosticating the outcome of SNF stays in patients with HF and possibly in other conditions based on one’s level of social connection.

The SCI is a unique tool designed for the nursing home setting. Many other tools have been used to characterize social isolation and loneliness in nursing homes, a topic that garnered special interest especially during the COVID-19 pandemic. These tools are largely reliant on self-report and calculate the number of objective connections that someone has or ask about subjective feelings of loneliness. The SCI can identify nursing home residents who exhibit behaviors detrimental to social connection, which can allow for real-time detection and intervention within the nursing home setting. Future studies should include self-report measures of social isolation and loneliness along with the SCI to assess concordance and to generate a provider- and patient-centered measure of social connectedness in nursing homes.

We also examined the raw proportions of unsuccessful discharge outcomes (death, remaining in the nursing home, and discharge to a noncommunity setting) and found that each type of unsuccessful discharge occurred at a higher rate in persons with low social connectedness than in persons with high social connectedness. This observation held in the stratum of the sample with AD/ADRD and the stratum without AD/ADRD. The rate of successful discharge in our cohort (37.7%) was slightly lower than a comparison cohort of 155,440 Medicare beneficiaries with HF who were discharged from SNF to home (43.4%).^34^ The successful discharge rate in the SCI = 5 group approximated the comparison cohort, whereas the rate of the SCI < 5 group was markedly lower (24.7%). From these data, it does not appear that any one type of unsuccessful discharge was driving the observed association between higher social connectedness and higher adjusted risk of successful discharge. It also appears that lower social connectedness is associated with markedly lower successful discharge rates compared with similar studies in the literature.

### Limitations

This study has several limitations. First, our observational study could not determine whether social connectedness directly caused successful discharge, whether it mediated a causal relationship between underlying health characteristics and the outcome of successful discharge, or whether social connectedness is a marker of beneficial health characteristics that are indirectly associated with successful discharge. We did not have information regarding social connectedness before admission, which limits our ability to describe a causal relationship between social connectedness and HF outcomes. As such, we assert that high social connectedness signifies a better prognosis for successful discharge than low social connectedness without positing a specific causal pathway. This observation is still potentially useful to clinicians engaged in discharge planning and rehabilitation after HF hospitalization. An additional limitation is that our study cohort consisted mostly of White men, which limits the generalizability of our findings to less homogeneous populations. Also, our measurement of social connectedness using an MDS-based scale is subject to the inherent limitations of the MDS, a regulation-required assessment instrument subject to the biases of the individuals who complete the assessments and the reporting institutions. Eighty-nine percent89% of veterans had high SCI, which may reflect a ceiling effect of the MDS-derived measure and limits our conclusions due to limited variability.

## Conclusions and Implications

We found that higher levels of social connectedness within the postacute SNF environment were associated with successful discharge in a cohort of veterans hospitalized for HF, and that lower social connectedness was associated with a higher rate of remaining in the SNF after 90 days. These findings were consistent in AD/ADRD and non-AD/ADRD cohorts and highlight an opportunity to improve nursing home discharge outcomes by facilitating social connection among residents through one-to-one personal contact, group activity, and increasing social access.^35,36^ The SCI may serve as a tool to identify residents at risk of future unsuccessful discharge and in need of more extensive SNF care, possibly reducing readmissions. Clinicians engaged in discharge planning for patients with HF in hospital and SNF settings should consider that higher social connectedness is associated with successful discharge home. Future research is needed on interventions to address low social connectedness in persons hospitalized for HF, especially when ADRD is also present.

## Supplementary Material

Supplemental Materials

Supplementary data related to this article can be found online at https://doi.org/10.1016/j.jamda.2025.105824.

## Figures and Tables

**Table 1:** Cohort Demographics

Variable	Overall (n = 29,725)	Low Social Connectedness (n = 3203)	High Social Connectedness (n = 26522)

Age, mean, SD	77.7 (10.3)	77.4 (10.2)	77.8 (10.3)
Male, n (%)	28,946 (97.4)	3127 (97.6)	25,819 (97.3)
Race, n (%)			
White	21,984 (73.8)	2392 (74.7)	19,556 (73.7)
Black	5604 (18.9)	566 (17.7)	5038 (19.0)
Hispanic	2109 (7.1)	239 (7.5)	1870 (7.1)
ADRD	9962 (33.5)	1479 (46.2)	8483 (32.0)
Elixhauser Comorbidity Index, mean (SD)	8.2 (2.6)	8.2 (2.6)	8.2 (2.6)
EF, n (%)			
0–40%	11,227 (37.8)	1251 (39.1)	9976 (37.6)
40%–50%	5410 (18.2)	564 (17.6)	4846 (18.3)
≥50%	11,276 (37.9)	1144 (35.7)	10,132 (38.2)
Prior year HF admission, n (%)	8449 (28.4)	934 (29.2)	7515 (28.3)
Smoking history, n (%)			
Prior smoker	24,822 (83.5)	2678 (83.6)	22,144 (83.5)
Never smoker	3579 (12.0)	393 (12.3)	3186 (12.0)
Moderate to severe pain level, n (%)	8661 (29.1)	939 (29.3)	7722 (29.1)
MDS 3.0 CFS, n (%)			
Intact	17,702 (59.6)	1257 (39.2)	16,445 (62.0)
Mild impairment	7375 (24.8)	908 (28.3)	6467 (24.4)
Moderate impairment	3905 (13.1)	857 (26.8)	3048 (11.5)
Severe impairment	483 (1.6)	138 (4.3)	345 (1.3)
Life expectancy <6 mo, n (%)	281 (0.9)	47 (1.5)	234 (0.9)
