# Supplementary material for: Social Connectedness and Successful Nursing Home Discharge After Heart Failure Hospitalization
Source: J Am Med Dir Assoc. Author manuscript; Available in PMC 2026 Apr 1. (PMC13041037; doi:10.1016/j.jamda.2025.105824)
Supplement: Supplemental Materials [file NIHMS2152275-supplement-Supplemental_Materials.docx]

**Supplemental File**

| **Page** | **Contents** |
| --- | --- |
| 2 | Table S1: International Classification of Disease 10 codes in the Veterans Health Administration Dementia Diagnostic Code List |
| 6 | Table S2: International Classification of Disease 9 codes in the Veterans Health Administration Dementia Diagnostic Code List |

**Table S1:** International Classification of Disease 10 codes in the Veterans Health Administration Dementia Diagnostic Code List

| **ICD10 Code** | **ICD10 Description** |
| --- | --- |
| A81.00 | CREUTZFELDT-JAKOB DISEASE, UNSPECIFIED |
| A81.01 | VARIANT CREUTZFELDT-JAKOB DISEASE |
| A81.09 | OTHER CREUTZFELDT-JAKOB DISEASE |
| A81.2 | PROGRESSIVE MULTIFOCAL LEUKOENCEPHALOPATHY |
| A81.82 | GERSTMANN-STRAUSSLER-SCHEINKER SYNDROME |
| A81.89 | OTHER ATYPICAL VIRUS INFECTIONS OF CENTRAL NERVOUS SYSTEM |
| A81.9 | ATYPICAL VIRUS INFECTION OF CENTRAL NERVOUS SYSTEM, UNSPECIFIED |
| F01.50 | VASCULAR DEMENTIA WITHOUT BEHAVIORAL DISTURBANCE, PSYCHOTIC DISTURBANCE, MOOD DISTURBANCE, AND ANXIETY |
| F01.511 | VASCULAR DEMENTIA, UNSPECIFIED SEVERITY, WITH AGITATION |
| F01.518 | VASCULAR DEMENTIA, UNSPECIFIED SEVERITY, WITH OTHER BEHAVIORAL DISTURBANCE |
| F01.52 | VASCULAR DEMENTIA, UNSPECIFIED SEVERITY, WITH PSYCHOTIC DISTURBANCE |
| F01.53 | VASCULAR DEMENTIA, UNSPECIFIED SEVERITY, WITH MOOD DISTURBANCE |
| F01.54 | VASCULAR DEMENTIA, UNSPECIFIED SEVERITY, WITH ANXIETY |
| F01.A0 | VASCULAR DEMENTIA, MILD, WITHOUT BEHAVIORAL DISTURBANCE, PSYCHOTIC DISTURBANCE, MOOD DISTURBANCE, AND ANXIETY |
| F01.A11 | VASCULAR DEMENTIA, MILD, WITH AGITATION |
| F01.A18 | VASCULAR DEMENTIA, MILD, WITH OTHER BEHAVIORAL DISTURBANCE |
| F01.A2 | VASCULAR DEMENTIA, MILD, WITH PSYCHOTIC DISTURBANCE |
| F01.A3 | VASCULAR DEMENTIA, MILD, WITH MOOD DISTURBANCE |
| F01.A4 | VASCULAR DEMENTIA, MILD, WITH ANXIETY |
| F01.B0 | VASCULAR DEMENTIA, MODERATE, WITHOUT BEHAVIORAL DISTURBANCE, PSYCHOTIC DISTURBANCE, MOOD DISTURBANCE, AND ANXIETY |
| F01.B11 | VASCULAR DEMENTIA, MODERATE, WITH AGITATION |
| F01.B18 | VASCULAR DEMENTIA, MODERATE, WITH OTHER BEHAVIORAL DISTURBANCE |
| F01.B2 | VASCULAR DEMENTIA, MODERATE, WITH PSYCHOTIC DISTURBANCE |
| F01.B3 | VASCULAR DEMENTIA, MODERATE, WITH MOOD DISTURBANCE |
| F01.B4 | VASCULAR DEMENTIA, MODERATE, WITH ANXIETY |
| F01.C0 | VASCULAR DEMENTIA, SEVERE, WITHOUT BEHAVIORAL DISTURBANCE, PSYCHOTIC DISTURBANCE, MOOD DISTURBANCE, AND ANXIETY |
| F01.C11 | VASCULAR DEMENTIA, SEVERE, WITH AGITATION |
| F01.C18 | VASCULAR DEMENTIA, SEVERE, WITH OTHER BEHAVIORAL DISTURBANCE |
| F01.C2 | VASCULAR DEMENTIA, SEVERE, WITH PSYCHOTIC DISTURBANCE |

| **ICD10 Code** | **ICD10 Description** |
| --- | --- |
| F01.C3 | VASCULAR DEMENTIA, SEVERE, WITH MOOD DISTURBANCE |
| F01.C4 | VASCULAR DEMENTIA, SEVERE, WITH ANXIETY |
| F02.80 | DEMENTIA IN OTHER DISEASES CLASSIFIED ELSEWHERE WITHOUT BEHAVIORAL DISTURBANCE, PSYCHOTIC DISTURBANCE, MOOD DISTURBANCE, AND ANXIETY |
| F02.811 | DEMENTIA IN OTHER DISEASES CLASSIFIED ELSWHERE, UNSPECIFIED SEVERITY, WITH AGITATION |
| F02.818 | DEMENTIA IN OTHER DISEASES CLASSIFIED ELSWHERE, UNSPECIFIED SEVERITY, WITH OTHER BEHAVIORAL DISTURBANCE |
| F02.82 | DEMENTIA IN OTHER DISEASES CLASSIFIED ELSWHERE, UNSPECIFIED SEVERITY, WITH PSYCHOTIC DISTURBANCE |
| F02.83 | DEMENTIA IN OTHER DISEASES CLASSIFIED ELSWHERE, UNSPECIFIED SEVERITY, WITH MOOD DISTURBANCE |
| F02.84 | DEMENTIA IN OTHER DISEASES CLASSIFIED ELSWHERE, UNSPECIFIED SEVERITY, WITH ANXIETY |
| F02.A0 | DEMENTIA IN OTHER DISEASES CLASSIFIED ELSWHERE, MILD, WITHOUT BEHAVIORAL DISTURBANCE, PSYCHOTIC DISTURBANCE, MOOD DISTURBANCE, AND ANXIETY |
| F02.A11 | DEMENTIA IN OTHER DISEASES CLASSIFIED ELSWHERE, MILD, WITH AGITATION |
| F02.A18 | DEMENTIA IN OTHER DISEASES CLASSIFIED ELSWHERE, MILD, WITH OTHER BEHAVIORAL DISTURBANCE |
| F02.A2 | DEMENTIA IN OTHER DISEASES CLASSIFIED ELSWHERE, MILD, WITH PSYCHOTIC DISTURBANCE |
| F02.A3 | DEMENTIA IN OTHER DISEASES CLASSIFIED ELSWHERE, MILD, WITH MOOD DISTURBANCE |
| F02.A4 | DEMENTIA IN OTHER DISEASES CLASSIFIED ELSWHERE, MILD, WITH ANXIETY |
| F02.B0 | DEMENTIA IN OTHER DISEASES CLASSIFIED ELSWHERE, MODERATE, WITHOUT BEHAVIORAL DISTURBANCE, PSYCHOTIC DISTURBANCE, MOOD DISTURBANCE, AND ANXIETY |
| F02.B11 | DEMENTIA IN OTHER DISEASES CLASSIFIED ELSWHERE, MODERATE, WITH AGITATION |
| F02.B18 | DEMENTIA IN OTHER DISEASES CLASSIFIED ELSWHERE, MODERATE, WITH OTHER BEHAVIORAL DISTURBANCE |
| F02.B2 | DEMENTIA IN OTHER DISEASES CLASSIFIED ELSWHERE, MODERATE, WITH PSYCHOTIC DISTURBANCE |
| F02.B3 | DEMENTIA IN OTHER DISEASES CLASSIFIED ELSWHERE, MODERATE, WITH MOOD DISTURBANCE |
| F02.B4 | DEMENTIA IN OTHER DISEASES CLASSIFIED ELSWHERE, MODERATE, WITH ANXIETY |
| F02.C0 | DEMENTIA IN OTHER DISEASES CLASSIFIED ELSWHERE, SEVERE, WITHOUT BEHAVIORAL DISTURBANCE, PSYCHOTIC DISTURBANCE, MOOD DISTURBANCE, AND ANXIETY |
| F02.C11 | DEMENTIA IN OTHER DISEASES CLASSIFIED ELSWHERE, SEVERE, WITH AGITATION |
| F02.C18 | DEMENTIA IN OTHER DISEASES CLASSIFIED ELSWHERE, SEVERE, WITH OTHER BEHAVIORAL DISTURBANCE |
| F02.C2 | DEMENTIA IN OTHER DISEASES CLASSIFIED ELSWHERE, SEVERE, WITH PSYCHOTIC DISTURBANCE |

| **ICD10 Code** | **ICD10 Description** |
| --- | --- |
| F02.C3 | DEMENTIA IN OTHER DISEASES CLASSIFIED ELSWHERE, SEVERE, WITH MOOD DISTURBANCE |
| F02.C4 | DEMENTIA IN OTHER DISEASES CLASSIFIED ELSWHERE, SEVERE, WITH ANXIETY |
| F03.90 | UNSPECIFIED DEMENTIA WITHOUT BEHAVIORAL DISTURBANCE, PSYCHOTIC DISTURBANCE, MOOD DISTURBANCE, AND ANXIETY |
| F03.911 | UNSPECIFIED DEMENTIA, UNSPECIFIED SEVERITY, WITH AGITATION |
| F03.918 | UNSPECIFIED DEMENTIA, UNSPECIFIED SEVERITY, WITH OTHER BEHAVIROAL DISTURBANCE |
| F03.92 | UNSPECIFIED DEMENTIA, UNSPECIFIED SEVERITY, WITH PSYCHOTIC DISTURBANCE |
| F03.93 | UNSPECIFIED DEMENTIA, UNSPECIFIED SEVERITY, WITH MOOD DISTURBANCE |
| F03.94 | UNSPECIFIED DEMENTIA, UNSPECIFIED SEVERITY, WITH ANXIETY |
| F03.A0 | UNSPECIFIED DEMENTIA, MILD, WITHOUT BEHAVIORAL DISTURBANCE, PSYCHOTIC DISTURBANCE, MOOD DISTURBANCE, AND ANXIETY |
| F03.A11 | UNSPECIFIED DEMENTIA, MILD, WITH AGITATION |
| F03.A18 | UNSPECIFIED DEMENTIA, MILD, WITH OTHER BEHAVIORAL DISTURBANCE |
| F03.A2 | UNSPECIFIED DEMENTIA, MILD, WITH PSYCHOTIC DISTURBANCE |
| F03.A3 | UNSPECIFIED DEMENTIA, MILD, WITH MOOD DISTURBANCE |
| F03.A4 | UNSPECIFIED DEMENTIA, MILD, WITH ANXIETY |
| F03.B0 | UNSPECIFIED DEMENTIA, MODERATE, WITHOUT BEHAVIORAL DISTURBANCE, PSYCHOTIC DISTURBANCE, MOOD DISTURBANCE, AND ANXIETY |
| F03.B11 | UNSPECIFIED DEMENTIA, MODERATE, WITH AGITATION |
| F03.B18 | UNSPECIFIED DEMENTIA, MODERATE, WITH OTHER BEHAVIORAL DISTURBANCE |
| F03.B2 | UNSPECIFIED DEMENTIA, MODERATE, WITH PSYCHOTIC DISTURBANCE |
| F03.B3 | UNSPECIFIED DEMENTIA, MODERATE, WITH MOOD DISTURBANCE |
| F03.B4 | UNSPECIFIED DEMENTIA, MODERATE, WITH ANXIETY |
| F03.C0 | UNSPECIFIED DEMENTIA, SEVERE, WITHOUT BEHAVIORAL DISTURBANCE, PSYCHOTIC DISTURBANCE, MOOD DISTURBANCE, AND ANXIETY |
| F03.C11 | UNSPECIFIED DEMENTIA, SEVERE, WITH AGITATION |
| F03.C18 | UNSPECIFIED DEMENTIA, SEVERE, WITH OTHER BEHAVIORAL DISTURBANCE |
| F03.C2 | UNSPECIFIED DEMENTIA, SEVERE, WITH PSYCHOTIC DISTURBANCE |
| F03.C3 | UNSPECIFIED DEMENTIA, SEVERE, WITH MOOD DISTURBANCE |
| F03.C4 | UNSPECIFIED DEMENTIA, SEVERE, WITH ANXIETY |
| F10.27 | ALCOHOL DEPENDENCE WITH ALCOHOL-INDUCED PERSISTING DEMENTIA |
| F10.97 | ALCOHOL USE, UNSPECIFIED WITH ALCOHOL-INDUCED PERSISTING DEMENTIA |
| F13.27 | SEDATIVE, HYPNOTIC OR ANXIOLYTIC DEPENDENCE WITH SEDATIVE, HYPNOTIC OR ANXIOLYTIC-INDUCED PERSISTING DEMENTIA |
| F13.97 | SEDATIVE, HYPNOTIC OR ANXIOLYTIC USE, UNSPECIFIED WITH SEDATIVE, HYPNOTIC OR ANXIOLYTIC-INDUCED PERSISTING DEMENTIA |
| F18.17 | INHALANT ABUSE WITH INHALANT-INDUCED DEMENTIA |
| **ICD10 Code** | **ICD10 Description** |
| F18.27 | INHALANT DEPENDENCE WITH INHALANT-INDUCED DEMENTIA |
| F18.97 | INHALANT USE, UNSPECIFIED WITH INHALANT-INDUCED PERSISTING DEMENTIA |
| F19.17 | OTHER PSYCHOACTIVE SUBSTANCE ABUSE WITH PSYCHOACTIVE SUBSTANCE-INDUCED PERSISTING DEMENTIA |
| F19.27 | OTHER PSYCHOACTIVE SUBSTANCE DEPENDENCE WITH PSYCHOACTIVE SUBSTANCE-INDUCED PERSISTING DEMENTIA |
| F19.97 | OTHER PSYCHOACTIVE SUBSTANCE USE, UNSPECIFIED WITH PSYCHOACTIVE SUBSTANCE-INDUCED PERSISTING DEMENTIA |
| G23.1 | PROGRESSIVE SUPRANUCLEAR OPHTHALMOPLEGIA [STEELE-RICHARDSON-OLSZEWSKI] |
| G30.0 | ALZHEIMER'S DISEASE WITH EARLY ONSET |
| G30.1 | ALZHEIMER'S DISEASE WITH LATE ONSET |
| G30.8 | OTHER ALZHEIMER'S DISEASE |
| G30.9 | ALZHEIMER'S DISEASE, UNSPECIFIED |
| G31.01 | PICK'S DISEASE |
| G31.09 | OTHER FRONTOTEMPORAL DEMENTIA |
| G31.83 | DEMENTIA WITH LEWY BODIES |
| G90.3 | MULTI-SYSTEM DEGENERATION OF THE AUTONOMIC NERVOUS SYSTEM |

**Table S2:** International Classification of Disease 9 codes in the Veterans Health Administration Dementia Diagnostic Code List

| **ICD9 Code** | **ICD9 Description** |
| --- | --- |
| 046.11 | VARIANT CREUTZFELDT-JAKOB DISEASE |
| 046.19 | OTHER AND UNSPECIFIED CREUTZFELDT-JAKOB DISEASE |
| 046.3 | PROGRESSIVE MULTIFOCAL LEUKOENCEPHALOPATHY |
| 046.71 | GERSTMANN-STRAUSSLER-SCHEINKER SYNDROME |
| 046.79 | OTHER AND UNSPECIFIED PRION DISEASE OF CENTRAL NERVOUS SYSTEM |
| 046.9 | UNSPECIFIED SLOW VIRUS INFECTION OF CENTRAL NERVOUS SYSTEM |
| 290.0 | SENILE DEMENTIA, UNCOMPLICATED |
| 290.10 | PRESENILE DEMENTIA, UNCOMPLICATED |
| 290.11 | PRESENILE DEMENTIA WITH DELIRIUM |
| 290.12 | PRESENILE DEMENTIA WITH DELUSIONAL FEATURES |
| 290.13 | PRESENILE DEMENTIA WITH DEPRESSIVE FEATURES |
| 290.20 | SENILE DEMENTIA WITH DELUSIONAL FEATURES |
| 290.21 | SENILE DEMENTIA WITH DEPRESSIVE FEATURES |
| 290.3 | SENILE DEMENTIA WITH DELIRIUM |
| 290.40 | VASCULAR DEMENTIA, UNCOMPLICATED |
| 290.41 | VASCULAR DEMENTIA, WITH DELIRIUM |
| 290.42 | VASCULAR DEMENTIA, WITH DELUSIONS |
| 290.43 | VASCULAR DEMENTIA, WITH DEPRESSED MOOD |
| 291.1 | ALCOHOL-INDUCED PERSISTING AMNESTIC DISORDER |
| 291.2 | ALCOHOL-INDUCED PERSISTING DEMENTIA |
| 292.82 | DRUG-INDUCED PERSISTING DEMENTIA |
| 294.1 | DEMENTIA IN CONDITIONS CLASSIFIED ELSEWHERE |
| 294.10 | DEMENTIA IN CONDITIONS CLASSIFIED ELSEWHERE WITHOUT BEHAVIORAL DISTURBANCE |
| 294.11 | DEMENTIA IN CONDITIONS CLASSIFIED ELSEWHERE WITH BEHAVIORAL DISTURBANCE |
| 294.20 | DEMENTIA, UNSPECIFIED, WITHOUT BEHAVIORAL DISTURBANCE |
| 294.21 | DEMENTIA, UNSPECIFIED, WITH BEHAVIORAL DISTURBANCE |
| 331.0 | ALZHEIMER'S DISEASE |
| 331.11 | PICK'S DISEASE |
| 331.19 | OTHER FRONTOTEMPORAL DEMENTIA |
| 331.82 | DEMENTIA WITH LEWY BODIES |
